# Vascular Shunt for Small Vessel Trauma in a Polytrauma Patient

**DOI:** 10.7759/cureus.9150

**Published:** 2020-07-12

**Authors:** Anupam K Gupta, Monica I Burgos, Faris Azar, Mario Rueda, Nir Hus

**Affiliations:** 1 Minimally Invasive Surgery, University of Miami Hospital, Miami, USA; 2 Internal Medicine, Universidad Autonoma de Guadalajara, Guadalajara, MEX; 3 Surgery, St. Mary's Medical Center, West Palm Beach, USA; 4 Trauma and Acute Care Surgery, St. Mary's Hospital, Boca Raton, USA; 5 Surgery, Delray Medical Center, Delray Beach, USA; 6 Surgery, Florida Atlantic University, Boca Raton, USA

**Keywords:** vascular shunt, small vessel, polytrauma, ulnar artery shunt

## Abstract

A 15-year-old male patient presented with multiple gunshot injuries. The patient underwent emergency lifesaving exploratory laparotomy and subsequently needed repair of his left upper limb ulnar artery injury. A shunt helped restore blood flow to the hand in a setting of damage control surgery in an exsanguinating patient with an ulnar artery injury having a massive disruption. After resuscitation, the patient underwent definitive repair of the artery using a vein interposition graft.

## Introduction

In gunshot wounds, patients with polytrauma lifesaving procedures become a priority [1]. Evaluation of vascular injury of the limb starts with clinical examination to look for hard signs / soft signs of vascular involvement. Loss of previously present pulses is a hard sign of vascular involvement [2]. In situations of damage control surgery in exsanguinating patients, there is not enough time to perform a vascular reconstruction. We describe the use of a vascular shunt in a small vessel across a joint to restore blood flow to the hand temporarily. We are thereby not only able to save the patient from bleeding, but also save the hand.

## Case presentation

**Figure 1:**
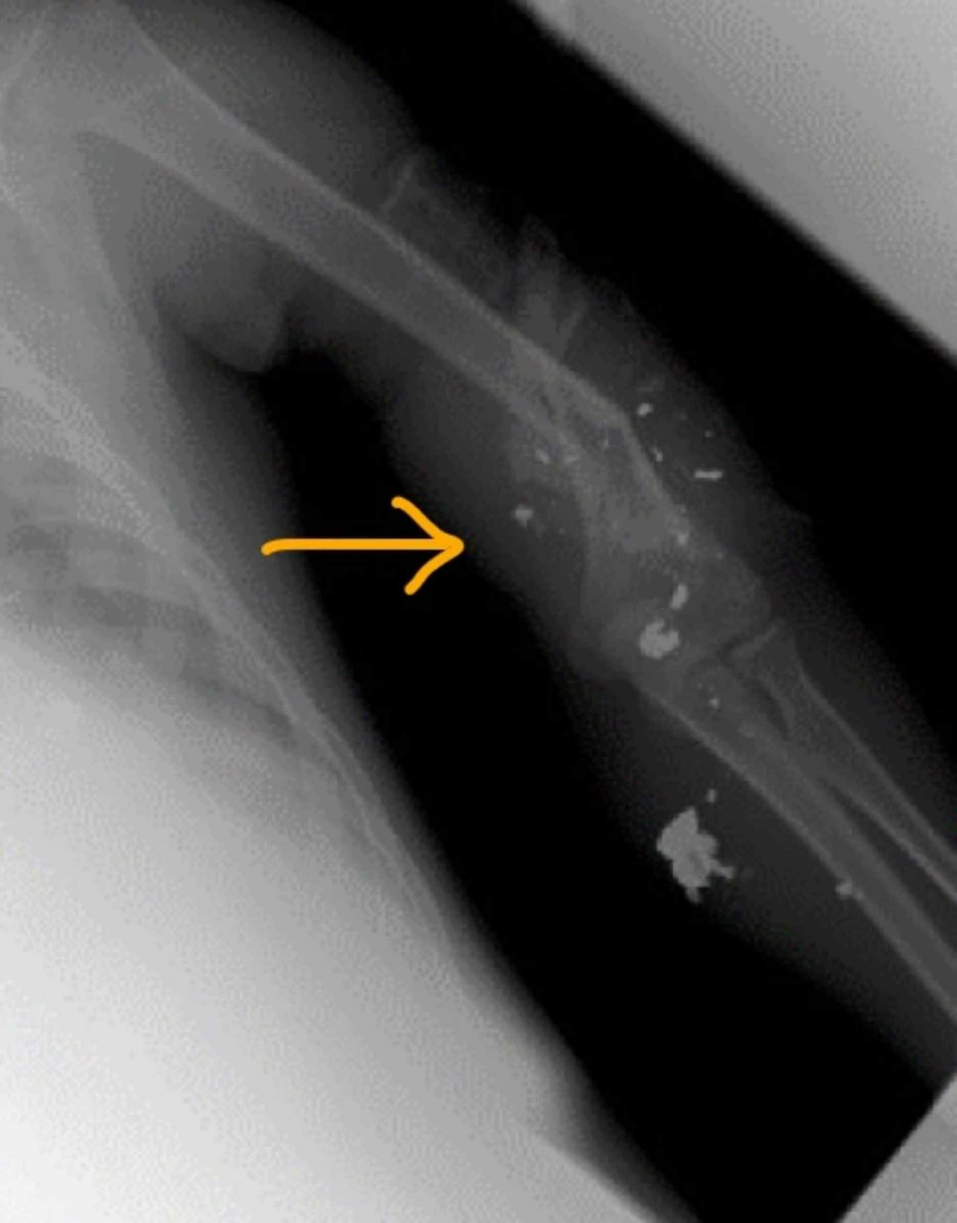
Left humerus bone fracture with bullet fragment

**Figure 2:**
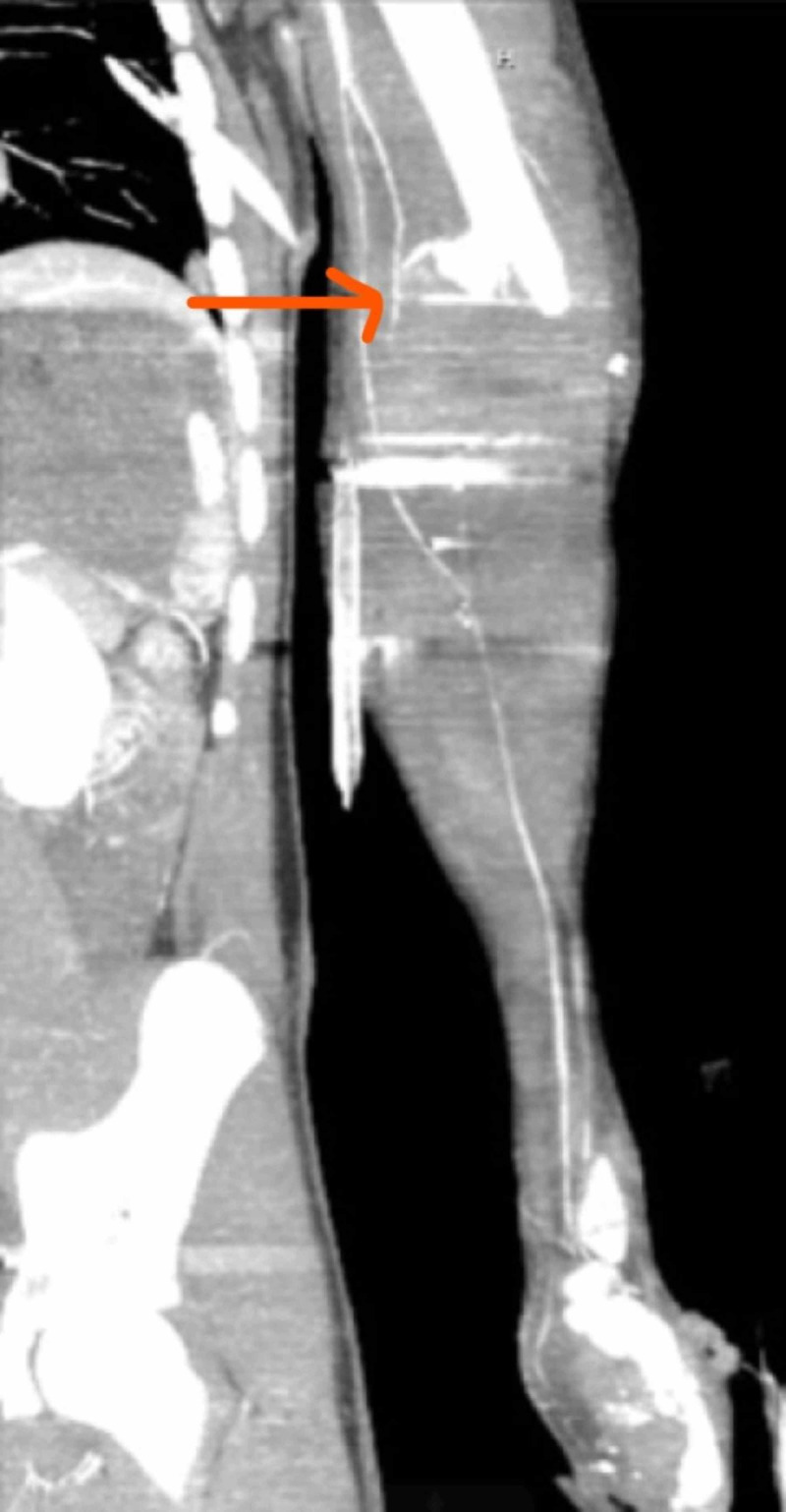
Left upper limb angiogram with high brachial artery bifurcation and flow in ulnar artery

A 15-year-old male patient presented to the trauma emergency room intubated in the field with multiple gunshot wounds. On the primary survey, the patient was tachycardic and intubated. The patient's heart rate ranged from 100 to 120 bpm, sinus tachycardia, and blood pressure 100/60 mm Hg. In the secondary survey, the patient had multiple gunshot wounds to the chest, abdomen, bilateral lower limbs, and left upper extremity. The patient had palpable pulses in the bilateral lower extremity and left upper extremity. In the trauma emergency room, the patient had left tube thoracostomy for a left hemothorax performed bedside. The patient underwent computed tomography imaging suggestive of pneumoperitoneum, an X-ray and angiogram of left upper extremity suggestive of left upper extremity comminuted humerus fracture and left upper limb ulnar artery injury (Figures 1, 2). The patient underwent damage control exploratory laparotomy. Diaphragmatic injury and bladder injuries were repaired primarily, small and large bowel left in discontinuity, and for pelvic wall bleeding, preperitoneal packing was performed. Abdomen fascia was left open with temporary closure performed using the wound VAC (vacuum-assisted device). For left humerus fracture with an expanding hematoma fasciotomy of the left upper limb was performed. On completion of the left upper limb's fasciotomy, there were no radial or ulnar pulses for Doppler signals.

**Figure 3:**
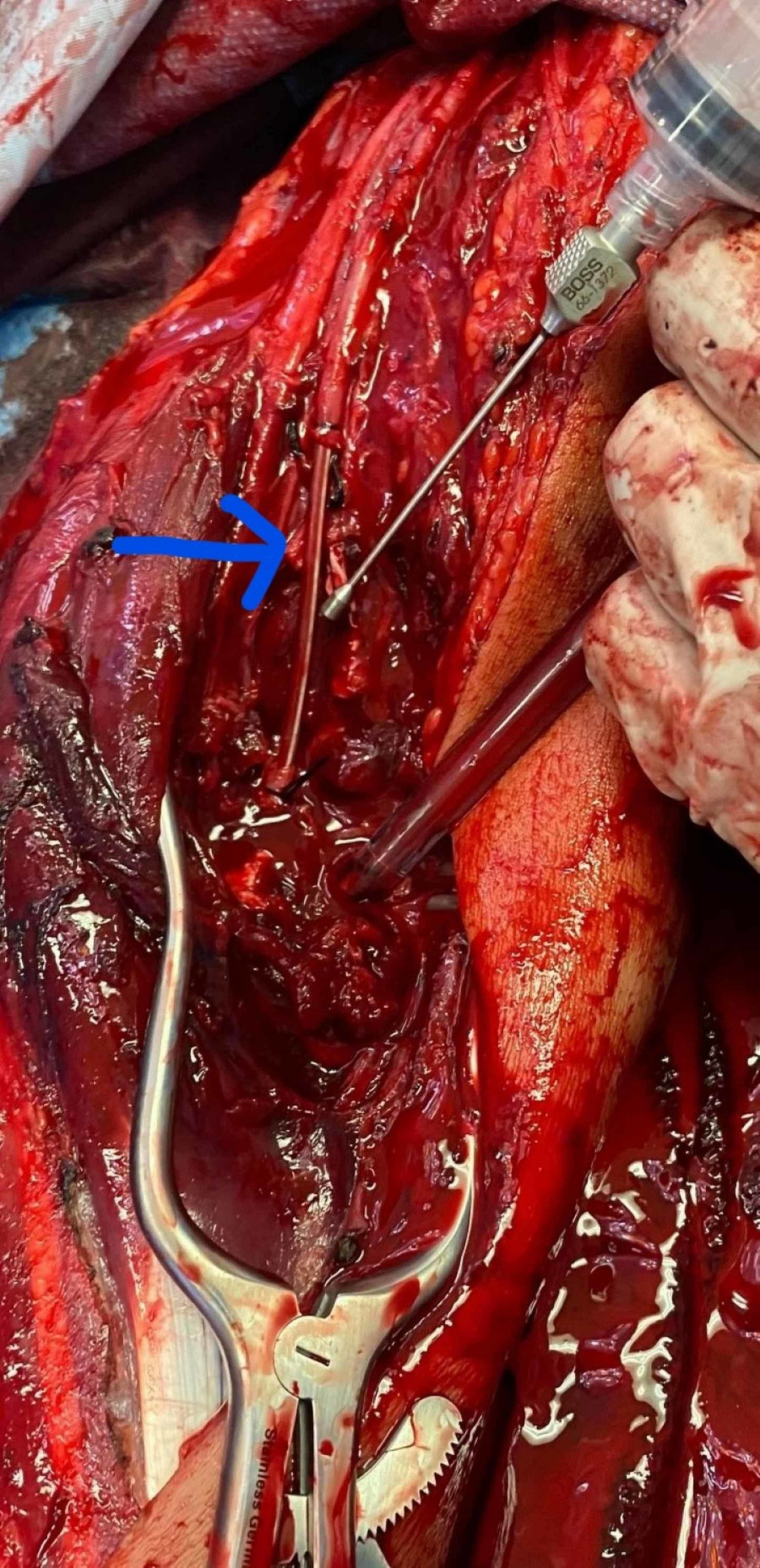
Argyl shunt in ulnar artery across elbow joint

**Figure 4:**
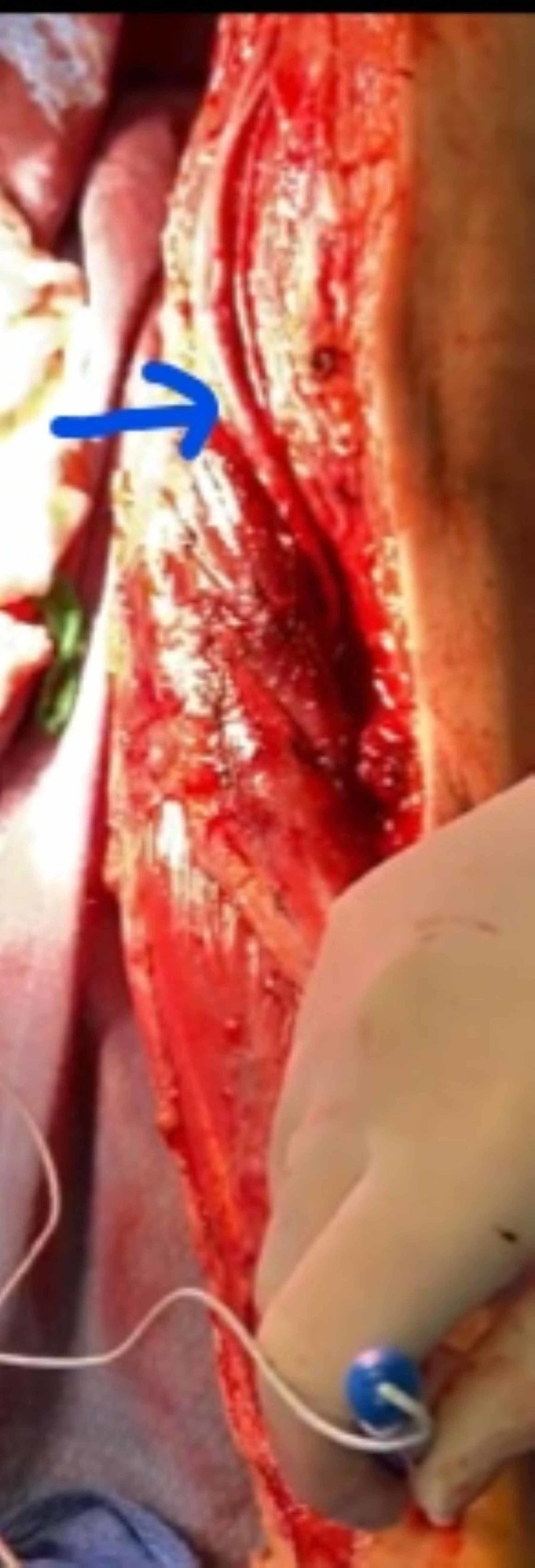
Basilic vein interposition draft with Doppler signals distally

An exploration of the brachial and ulnar artery revealed a disruption in the ulnar artery with a 5-cm arterial segment loss secondary to the gunshot and bullet fragments. An effort to mobilize the blood vessel did not permit a tension-free anastomosis. The artery was 2 mm in diameter. A Fogarty embolectomy showed good flow proximally, however weak backflow. At the time the patient had worsening acidosis and exsanguination, the ulnar artery was shunted with an argyle 8 Fr shunt (Figure 3). The patient had Doppler signals in the left upper extremity after the shunt. The patient had forearm and hand fasciotomy completed of his left upper limb. The patient underwent resuscitation followed by, angioembolization of the left internal iliac artery for pelvic bleed. Once hemoglobin was stable and base deficit corrected, the patient's left upper limb was re-explored, and the shunt removed and basilic vein interposition graft performed (Figure 4). The patient continued to have good pulses distally. The patient had an external fixation of his left humerus fracture, and a relook laparotomy to remove the pre-peritoneal packs placed earlier and perform bowel anastomosis. The patient received no anticoagulation, given his high risk of bleed in the pelvis and abdomen. The patient underwent surgeries for his wounds and went on to have complete vascular recovery from the interposition graft.

## Discussion

Polytrauma's most common causes are road traffic accidents, falls from heights, and bullet injuries. The most substantial toll of traumatic deaths occurs within the first hour following trauma [1].

Advanced trauma life support (ATLS) is a protocol developed to standardize the initial evaluation and management of injured patients and avoid omission of potentially lifesaving interventions. The first phase of ATLS is the primary survey, and it is a rapid evaluation of identifying life-threatening injuries [3]. The steps include evaluation and treatment of airway injuries (A, airway) followed by evaluation of respiratory dynamics (B, breathing), evaluation of the patient's hemodynamic status (C, circulation), and a neurologic assessment (D, disability) [3]. After completion of the airway and breathing evaluation, the assessment of circulation is next. After stabilizing the airway and breathing, an initial evaluation of the patient's circulatory status is done by palpating central pulses [4]. Any obvious hemorrhaging should be controlled by direct pressure if possible, and if needed, by applying tourniquets to the extremities. If manual compression fails to control hemorrhage, there is level III evidence to support a tourniquet until definitive repair [2]. Any patient presenting with pale, cold extremities, should be considered in shock until proven otherwise. After completing the primary survey, it is followed by the secondary survey, a detailed head-to-toe evaluation that identifies other injuries [5]. Damage control surgery is to restore normal physiology over restoring normal anatomy in the unstable, trauma patient. During exploratory laparotomy, it is essential to be cognizant of the lethal triad of hypothermia, acidosis, and coagulopathy [6]. This strategy aims to facilitate surgical control of hemorrhage and contamination, the potentially fatal problems at first look laparotomy, with secondary resuscitation followed by scheduled definitive surgery.

Common injuries resulting from polytrauma include vascular injuries. They have the potential to cause morbidity if not recognized in time. Literature states that vascular injuries are of five different types: 1. Intimal injuries (subintimal hematomas, flaps, disruptions), 2. Total wall defects with bleeding, hematomas, or pseudoaneurysm, 3. Total disruptions with bleeding and occlusions, 4. Arteriovenous fistulas, 5. Spasm [7]. Several diagnostic methods, including serial physical examination, local wound exploration, ultrasound, computerized tomography, diagnostic peritoneal lavage, diagnostic laparoscopy, may be used.

Vascular injuries are evaluated clinically by hard or soft signs. Hard signs are made up of distal circulatory deficit, active hemorrhage, rapidly expanding hematomas, absent pulses, pallor, paresthesia, pain, paralyzes, poikilothermia [2,8]. The soft signs are a history of arterial bleeding at the scene of injury, diminished distal unilateral pulse, small hematoma, neurological deficit, adjacent nerve injury, shock unexplained by other injuries, the proximity of penetrating wound to major vascular structures, abnormal flow velocity wave on Doppler examination, or abnormal ankle-brachial pressure index (ABI), <0.9 [2,8]. The presence or not of hard clinical signs decides whether the patient needs immediate surgical intervention. When signs are further ambivalent, investigations by calculating the ankle-brachial index, followed by imaging with CT angiography, help determine vascular status.

Patients with positive hard signs certainly require immediate operative intervention [7,9]. Time warrants necessary repair of an arterial injury within six hours before irreversible ischemia occurs [9]. Vascular reconstruction will often not be a priority, depending on the environmental conditions. First, the need to first stabilize the patient, fixate a fracture, or the need to perform damage control laparotomy, and other procedures is more important unless life-threatening hemorrhage occurs. The vascular injury becomes a priority, like in our patient needing control of bleeding from pelvis with preperitoneal packing [10]. In situations where the patient is undergoing damage control surgery, there is not enough time to perform an official vascular repair; thus hemostasis and revascularization become primarily dependent on damage control techniques, and temporary vascular shunts (TVS) for control of the vessel bleed is used [11]. Tourniquets or vascular clamps help to control proximal vessel bleeding initially [10]. Restoring blood flow in a timely and effective manner helps reduce the rate of amputations. Tourniquets for hemorrhage control, temporary shunts for early restoration of perfusion, and low threshold for fasciotomy are essential adjuncts to consider when facing delayed or complex upper extremity vascular injury [10].

Furthermore, a temporary vascular shunt can control the bleed of the vessel while other severe injuries are given priority. A TVS of the appropriate size is chosen and flushed with heparinized saline. It is crucial that the diameter of the TVS closely approximates that of the artery because, with size mismatch, the risk of rupture and hemorrhage is there [11]. The ability to temporize ongoing hemorrhage via proximal occlusion offers a distinct advantage for the rapid management of extremity injuries. After vessel repair, distal vascular status is evaluated, and the limb assessed for compartment syndrome. Early fasciotomy should liberally be applied when there has been prolonged ischemia or associated injuries [7]. The interval before definitive revascularization depends on the overall condition of the patient. In our patient, we waited for the stabilization of hemoglobin and corrected base deficit.

Approximately 95% of arterial injuries in the forearm are due to the penetrating injury [7]. Distal vessels such as the ulnar artery are small at baseline and prone to significant spasm in injured patients who are cold and in shock, limiting outflow and patency. In our patient, though we had a flow-on angiogram initially, we lost flow after the damage control surgery and fasciotomy [12]. The ulnar artery is usually the dominating vessel, and failure can lead to hand ischemia requiring amputation and was the reason for exploring the vessel in our patient. Also it was perceived at the time that it would be an easy exposure as we knew the level of injury on angiogram [7]. Classic arterial repair consists of either a direct suture repair, a vein graft, or a prosthetic graft. These techniques require one or two anastomoses, and the time needed for a repair is often inconsistent with the primary objectives of damage control that seek to limit the duration of surgery to less than 60 minutes [7]. Historically, this carries an extremity amputation rate of 27% after blunt trauma and 9% after penetrating trauma [13]. Shunts in smaller more-distal vessels have lower patency rates. Shunt tubes are of two categories: two-way and three-way types. The former includes Sundt, Javid, and Argyle shunts, and the latter Pruitt-Inahara, Brener, and Furui shunt [14]. Some commercial arterial shunts are too large for the distal brachial artery and ulnar arteries; thus, an appropriately sized shunt uses balances between maintaining patency and preventing an unwanted intimal injury [15]. The shunt used in our patient was small enough to be used effectively. The reduction in ischemia time may result in lower rates of compartment syndrome, nerve injury, and muscle loss, which may improve the overall quality of limb salvage and, in turn, prevent late amputation due to poor limb function [10]. A temporary vascular shunt typically remains in place while other procedures such as vein harvest or extremity fracture reduction are performed [15]. The brachial or basilic veins are favored for reconstruction because they lie within the exposures required to deal with colocated arterial injury and are easily protected with soft tissue coverage. Both military and civilian experience indicate that proximal extremity arterial shunts remain patent in between 85% and 95% of cases and do not negatively impact limb-salvage rates when used in proximal vessels [15].

Decision making in situations like this is very complex. Multiple team members, individuals, and specific variables influence the treatment algorithm, whether the damaged limb can be salvaged or needs an amputation [16]. In our case, intravascular shunting saved the hand of our patient. Management of these injuries is treated and evaluated on a case-by-case basis. When other injuries are involved, the management of traumatic vascular injuries becomes challenging to manage. As a team, we must prioritize the repairs based on the clinical presentation of the patient and their hemodynamic status. When vascular injuries are present in conjunction with life-threatening emergencies, controlling hemorrhage from a blood vessel may take initial priority; however, sacrificing a limb to preserve life is a well-established assertion [10].

## Conclusions

In an emergency damage control situation in an exsanguinating patient, the vascular shunt is an excellent temporizing technique for repairing small vessel injury.
